# Analysis of circulating tumor DNA during checkpoint inhibition in metastatic melanoma using a tumor-agnostic panel

**DOI:** 10.1097/CMR.0000000000000903

**Published:** 2023-06-12

**Authors:** Judit Kisistók, Ditte Sigaard Christensen, Mads Heilskov Rasmussen, Lone Duval, Ninna Aggerholm-Pedersen, Adam Andrzej Luczak, Boe Sandahl Sorensen, Martin Roelsgaard Jakobsen, Trine Heide Oellegaard, Nicolai Juul Birkbak

**Affiliations:** a Department of Molecular Medicine, Aarhus University Hospital; b Department of Clinical Medicine, Aarhus University; c Bioinformatics Research Center, Aarhus University; d Department of Oncology, Aarhus University Hospital, Aarhus; e Department of Oncology, Goedstrup Hospital, Herning; f Department of Oncology, Aalborg University Hospital; g Department of Clinical-Biochemistry, Aarhus University Hospital; h Department of Biomedicine, Aarhus University, Aarhus, Denmark

**Keywords:** circulating tumor DNA, immunotherapy, melanoma

## Abstract

Immunotherapy has revolutionized treatment of patients diagnosed with metastatic melanoma, where nearly half of patients receive clinical benefit. However, immunotherapy is also associated with immune-related adverse events, which may be severe and persistent. It is therefore important to identify patients that do not benefit from therapy early. Currently, regularly scheduled CT scans are used to investigate size changes in target lesions to evaluate progression and therapy response. This study aims to explore if panel-based analysis of circulating tumor DNA (ctDNA) taken at 3-week intervals may provide a window into the growing cancer, can be used to identify nonresponding patients early, and determine genomic alterations associated with acquired resistance to checkpoint immunotherapy without analysis of tumor tissue biopsies. We designed a gene panel for ctDNA analysis and sequenced 4–6 serial plasma samples from 24 patients with unresectable stage III or IV melanoma and treated with first-line checkpoint inhibitors enrolled at the Department of Oncology at Aarhus University Hospital, Denmark. *TERT* was the most mutated gene found in ctDNA and associated with a poor prognosis. We detected more ctDNA in patients with high metastatic load, which indicates that more aggressive tumors release more ctDNA into the bloodstream. Although we did not find evidence of specific mutations associated with acquired resistance, we did demonstrate in this limited cohort of 24 patients that untargeted, panel-based ctDNA analysis has the potential to be used as a minimally invasive tool in clinical practice to identify patients where the benefits of immunotherapy outweigh the drawbacks.

## Introduction

Advanced melanoma is an aggressive cancer type with an overall poor survival rate and limited response to traditional cancer therapy regimes such as chemotherapy. Over the past decade, immunotherapy has entered the clinic and has now become a cornerstone of the treatment of melanoma with remarkably improved overall survival (OS) rates [1]. Nevertheless, while almost half of the patients will benefit from the treatment and a considerable subset may even become long-term survivors, the remaining patients will experience little to no benefit from the therapy. Considering that immunotherapy is associated with potentially persisting adverse events for the patients, there remains an unmet need for understanding why some patients will benefit from immunotherapy while other patients will not. Currently, this remains unclear. Several studies have explored biomarkers that may predict response to immunotherapy. Potential biomarkers include high PDL1 expression for anti-PD1/PDL1 therapies, and high tumor mutational burden (TMB), both now approved by the United States Food and Drug Administration (FDA) as an indication for using immunotherapy unrelated to diagnosis [2]. Other biomarkers have been reported as associated with immunotherapy response, including clonal TMB [3], an inflammation gene expression signature [4], and a signature based on soluble PD-1 [5]. Additionally, somatic mutations of specific genes in the cancer cells have been found to influence the tumor microenvironment and the ability of tumor cells to evade the immune system, and hereby confer immunotherapy resistance [6]. These include inactivating mutations in *PTEN*, the third most frequently mutated gene in melanoma, which has been reported to be associated with resistance to checkpoint inhibition (CPI) in patients suffering from this disease [7].

Circulating tumor DNA (ctDNA) is defined as the fraction of cell-free DNA found in the blood and is derived from the tumor. In recent years ctDNA has been extensively studied as a noninvasive biomarker in multiple cancer types [8]. Analysis of ctDNA can identify tumor-specific mutations, which reflect the genetic composition of the entire tumor, and ctDNA levels have been shown to correlate with both tumor burden and clinical outcomes during treatment [9]. Targeted deep sequencing of ctDNA at different time points during immunotherapy for genes involved in cancer cells’ immune evasion may help resolve clonal diversity and identify the resistant clone. By timing molecular alterations with the onset of resistance, it may be possible to decipher one possible contributing factor of resistance to immunotherapy. Several studies have found that patients with detectable ctDNA prior to treatment had worse progression-free survival (PFS) and worse OS than patients with undetectable ctDNA. Moreover, changes in ctDNA levels have been found to correlate with radiologic response, and a decrease in ctDNA level during therapy was shown to be associated with response and longer PFS and OS [10–18]. Taking ctDNA assays into the clinic has also faced challenges due to sensitivity limitations, particularly in patients with metastases at sites protected by the organ blood barrier [19]. Nevertheless, the overall utility of ctDNA as a noninvasive biomarker with insights into tumor biology makes it a valuable tool for real-time monitoring of response during treatment.

In this prospective study, we aim to improve the understanding of the tumor biology that drives immunotherapy resistance in metastatic melanoma. We designed a clinical trial, where we hypothesized that patients diagnosed with melanoma and treated with immunotherapy would fall into three groups: those that respond initially and continue to respond (responders), those that fail to ever respond (innate resistance), and those that initially respond, but over time develop resistance (acquired resistance). By using a custom tumor-agnostic ctDNA panel to identify genomic alterations before, during, and after immunotherapy, we show how ctDNA levels remain low or undetectable in patients with therapy response, while it is present or increases in patients resistant to therapy. Additionally, by including known melanoma driver genes in the ctDNA panels, we demonstrate how it is possible to acquire novel insights into the biology behind response and resistance to immunotherapy.

## Materials and methods

### Patients

Clinical characteristics are summarized in Supplementary Table 1, Supplemental digital content 1, http://links.lww.com/MR/A326, and a full clinical table is found in Supplementary Table 2, Supplemental digital content 1, http://links.lww.com/MR/A326. Patients with unresectable, previously untreated stage III or IV melanoma who received systemic treatment with immune checkpoint inhibitors were eligible for the study. Key inclusion criteria were the absence of uveal melanoma, absence of another primary cancer, and no previous diagnosis of cancer. In total, we enrolled 24 patients with metastatic melanoma treated with first-line checkpoint inhibitors at Aarhus University Hospital in 2017. Ten patients received pembrolizumab at a dose of 2 mg/kg every 3 weeks and the remaining 14 patients received nivolumab 1 mg/kg plus ipilimumab 3 mg/kg every 3 weeks, followed by maintenance nivolumab 1 mg/kg. Median follow-up time was 794 days, range (63–2008).

### Disease characteristics and response assessment

Patient demographics and clinicopathologic features included: age, sex, performance status, metastatic sites at baseline, and lactate dehydrogenase (LDH). Elevated LDH level was defined as levels above 205 units/l (U/l) for patients below the age of 70 and above 255 U/l for patients above the age of 70. Diagnostic tumor biopsies were routinely screened for BRAFV600 status and PD-L1 expression level (</> 1%). Treatment responses were evaluated by PET/computed tomography (CT) scans of the chest, abdomen, and pelvis, and MRI in case of known brain metastases. We defined immunotherapy response groups as either responders (11/23 patients with long-lasting response to the treatment), resistance (9/23 patients with no response), and acquired resistance (4/23 patients with acquired resistance). The definition of acquired resistance encompassed patients that initially showed response on first-line therapy based upon at least one CT evaluation scan, but later progressed or died within 12 months of follow-up. Median time to progression or death for responder patients was 639 days (range 487–1362, 5/11 patients progressed or died during follow-up), 121 days for resistant patients (range 30–273, 9/9 patients progressed and died during follow-up), and 200 days for acquired resistance patients (range 152–334, 4/4 patients progressed or died during follow-up). Median follow-up time was 1893 days for responders (range 778–2008), 293 days for resistant patients (range 63–1166), and 453 days for acquired resistance patients (range 248–809). Additionally, we defined metastatic load groups as high (≥3 metastatic sites, 12/24) and low (<3 metastatic sites, 12/24). The number of metastatic sites was determined through CT scans at follow-up times. Survival status was evaluated at the end of the follow-up period (14/24 deceased, 10/24 alive).

### Sample collection and preparation

Peripheral blood samples (3 × 10 ml EDTA tubes, BDVacutainer, Plymouth, UK) were obtained at baseline (immediately before treatment initiation) and every 3–4 weeks during treatment for up to 1 year after treatment initiation. Plasma was isolated from peripheral blood samples within 2–3 h after blood collection by 1800 × g for 10 min at room temperature. Plasma was cryopreserved at −80 °C.

### Circulating-free DNA extraction

Circulating-free DNA was extracted from 4 ml plasma using the QIAamp Circulating Nucleic Acid Kit (Qiagen, Hilden, Germany) according to the manufacturer’s protocol. The isolated DNA was eluted in a 100 µl elution buffer and stored at −80 °C until analysis.

### Circulating tumor DNA analysis and sequencing

A custom gene panel for next-generation sequencing for ctDNA analysis was designed using Qiagen’s design services covering a total of 40 genes. The 40 genes chosen for the panel are known to be associated with cancer cells' susceptibility to immune attack (Supplementary Table 3, Supplemental digital content 1, http://links.lww.com/MR/A326). The panel covers a total of 150 000 base pairs of genomic content. Sequencing of the ctDNA panel was performed at 15 000x using an Illumina NovaSeq platform (San Diego, California, USA). In addition to the whole genes in the panel, the *TERT* promoter region was sequenced.

### Circulating tumor DNA variant calling and filtering

Variants were called using the Shearwater algorithm from the deepSNV R package [20,21]. Variants were called on a per-sample basis and the set of samples independent of the currently analyzed sample was used as background. Mutant allele frequency (MAF) and *P* values per position and alteration were calculated and the most significant alteration as well as the reference allele per position were identified. Only driver alterations that were significant (*P* ≤ 0.05) at any time point per patient were retained, the remaining alterations were excluded from further analysis. The retained variants were annotated using Annovar based on the hg38 reference genome. Variants were excluded as potential single nucleotide polymorphisms (SNPs) if their MAF exceeded 0.4, if ExAC or gnomAD values exceeded 0.01, or if they were marked as likely SNPs based on high (>0.1) and constant MAF over time. Driver mutations were defined as previously described [22], essentially based on detrimental mutations or frameshifts to known cancer genes as defined by the Catalogue of Somatic Mutations in Cancer (COSMIC) [23] cancer gene census [24]. In particular, the annotation was performed in the following manner:

1. A driver gene list was compiled from genes present in the COSMIC cancer gene census, as well as genes found in large pan-cancer studies [25].
2. If a gene was listed in the COSMIC cancer gene census as a tumor suppressor and two out of the three computational methods (Sift [26], Polyphen [27], and MutationTaster [28]) identify the variant as stop-gain or predicted deleterious, then it was annotated as a driver mutation.
3. If a gene was listed in the COSMIC cancer gene census as an oncogene and an exact match of the variant was found in COSMIC at least three times, the variant was again annotated as a driver variant.

To further reduce noise from SNPs and low-frequency subclonal mutations, only variants considered likely driver mutations were used in the analysis. A filtered variant table including all driver variants found significant in a given patient at least once is found in Supplementary Table 4, Supplemental digital content 1, http://links.lww.com/MR/A326.

### Sensitivity analysis

We performed in-silico benchmarking by running the Shearwater algorithm on a synthetic dataset. The test data was generated in the following manner:

1. A patient’s baseline sample was chosen randomly, and the count matrix was filtered for the BRAF V600E position. From the count matrix containing this position in 93 samples (the random patient’s baseline sample and all other samples from the remaining patients), the ranges of counts for A and T nucleotides were obtained for the forward and backward strands.
2. A test matrix was generated where the 93 rows corresponded to samples and the 10 columns corresponded to nucleotides (A, T, G, C, and X corresponding to deletion, forward, and backward strands).
3. Nucleotide A (columns 1 and 6) was chosen as reference, and nucleotide T (columns 2 and 7) was chosen as variant.
4. Using the count ranges obtained in Step 1, the counts were increased for the variant nucleotide T in each iteration, starting from 0 to the maximum observed variant count. The A nucleotide counts were set to the maximum observed reference count.
5. Each resulting count matrix was analyzed by Shearwater, yielding a *P* value for the variant. The MAFs were calculated by dividing the variant count by the corresponding row sum.

After summarizing each iteration, we report a median MAF limit of detection of 0.062 (interquartile range: 0.039–0.084, Supplementary Figure 1, Supplemental digital content 2, http://links.lww.com/MR/A327).

### Statistical analysis

Per-patient MAF was calculated by taking the mean MAF of variants annotated as likely driver mutations, per time point. OS was defined as the time from treatment initiation to the date of reported death due to any cause. Patients without disease progression or who were still alive at the last follow-up were censored at the last follow-up date (15 August 2021). All analysis was performed in R version 3.6.2, using Tidyverse [29] and ggpubr [30], scales [31], ggrepel [32] for visualizations. For significance testing, Wilcoxon test was used unless otherwise mentioned. *P* values less than 0.05 were considered significant. All *P* values are two-sided.

## Results

### Cohort overview

**Fig. 1:**
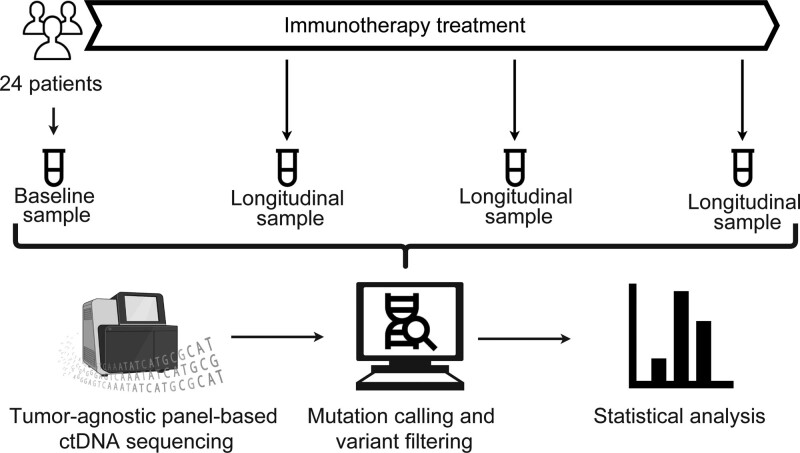
Study overview. Twenty-four patients with metastatic melanoma were enrolled in the study at Aarhus University Hospital in 2017. Prior to receiving systemic treatment with first-line checkpoint inhibitors, a baseline blood sample was taken. Subsequently, 10 patients were selected to receive treatment with Pembrolizumab and the remaining 14 patients received a combination of Nivolumab and Ipilimumab. Over the course of treatment, three additional blood samples were extracted. After cell-free DNA extraction from plasma, circulating tumor DNA was sequenced at 15000x depth using a custom gene panel consisting of 40 genes known to be associated with immunotherapy response. Mutations were called in the resulting ctDNA data by running the Shearwater algorithm on a per-sample basis, using the samples from independent patients as background. Following statistical analyses were performed in R.

We endeavored to investigate the utility of tracking response to immunotherapy and development of treatment resistance using a custom tumor-agnostic ctDNA panel. For this purpose, we collected plasma samples from a cohort of 24 patients with metastatic melanoma, starting first-line checkpoint immunotherapy with either pembrolizumab or with a combination of nivolumab and ipilimumab. From all patients, a baseline blood sample was analyzed, followed by additional blood samples drawn during treatment. From these, we purified and analyzed ctDNA using a custom panel and an in-house bioinformatics pipeline (Fig. 1). Patients were grouped into response (lasting response to immunotherapy, eleven patients), acquired resistance (initial response, then acquired resistance to immunotherapy, four patients), and resistance (no response to immunotherapy, nine patients).

### Circulating tumor DNA frequency shows no difference between response groups

**Fig. 2:**
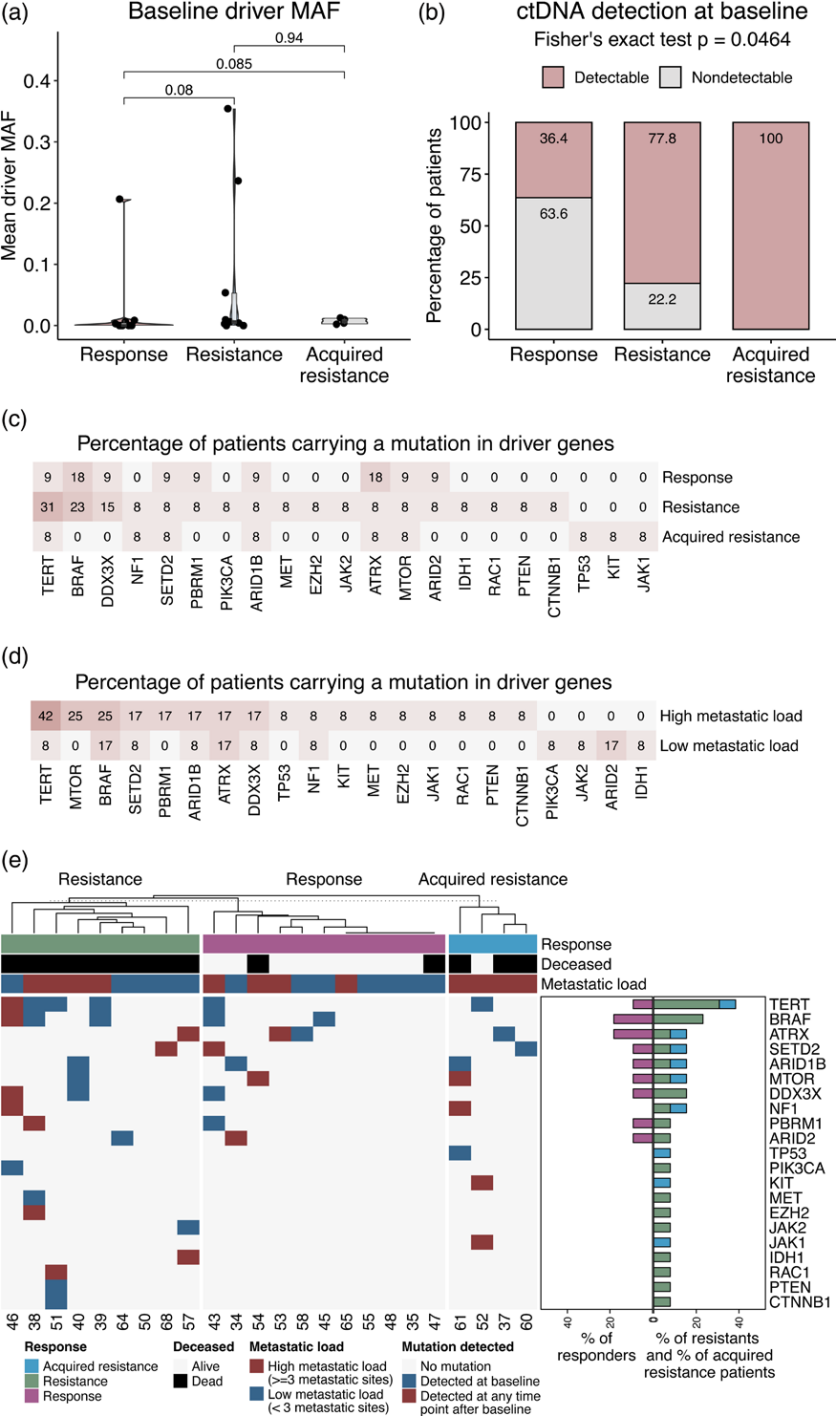
Mutation analysis. (a) Violinplots showing the mutant allele frequency at baseline. Values shown on the Y-axis are calculated by taking the mean MAF overall filtered variants per patient. X-axis and color show response category. (b) Stacked bar plots showing the percentage of patients per response category where ctDNA was detected at baseline. X-axis shows response category, Y-axis displays patient percentage, color corresponds to the detection status at baseline. (c) Heatmap showing the percentage of patients carrying mutations in panel genes. The percentages are calculated within categories. Color intensity corresponds to the percentage values. (d) Heatmap showing the percentage of patients carrying mutations in panel genes, split according to metastatic load categories. Visualization follows panel (c). (e) Heatmap-barplots showing the driver mutation profile of the cohort. Blue tiles indicate that a mutation was detected at baseline, red tiles indicate mutations that were not found at baseline but were detected at a later time point. Top annotations show patient characteristics: response category (pink: response, green: resistance, blue: acquired resistance), survival status (gray: alive, black: dead), and metastatic load (blue: low, <3 metastatic sites, red: high, ≥3 metastatic sites). Barplots on the side show the percentage of patients carrying a driver mutation per response category (pink: response, green: resistance, blue: acquired resistance). ctDNA, circulating tumor DNA; MAF, mutant allele frequency.

Relative to a tumor-informed ctDNA approach, our tumor-agnostic approach had the benefit of not requiring prior tumor DNA sequencing in order to call cancer mutations in plasma; however, this benefit comes with an increased risk of calling germline variants as potential tumor mutations. To minimize this, only variants considered likely cancer driver mutations were included in the downstream analysis (see methods). For all patients, we calculated the mean MAF for all driver-annotated variants found significant at baseline on a per-patient basis. For 9/24 patients where ctDNA detection was not possible at baseline, we set the mean MAF to 0. In our limited cohort of 24 patients, we observed no differences in baseline ctDNA MAF between patients with a response to treatment versus patients with no response to treatment (Fig. 2a); however, when comparing ctDNA detection status at baseline, we observed a significant difference between the three response categories, showing that resistant and acquired resistant patients released ctDNA at baseline with higher likelihood. (*P* = 0.0464, Fig. 2b). Additionally, when performing a survival analysis comparing patients with ctDNA detected at baseline to patients with no ctDNA detected, no significant difference was found, although a trend towards poor outcome for patients with ctDNA can be observed (*P* = 0.15, Supplementary Figure 2, Supplemental digital content 3, http://links.lww.com/MR/A328). Considering the limited size of the present cohort, it is likely that in a larger cohort, an association with outcome would be found.

We explored the association between ctDNA MAF and survival. We have found no significant difference in baseline MAF (*P* = 0.24) or ctDNA detection status at baseline (*P* = 0.403) when comparing patients who died during the study with those who remained alive (Supplementary Figure 3A and B, Supplemental digital content 4, http://links.lww.com/MR/A329). We then investigated whether ctDNA MAF might be associated with metastatic burden. Here, we observed that patients with a high metastatic load harbored significantly higher ctDNA MAF levels at baseline (*P* = 0.041, Supplementary Figure 4A, Supplemental digital content 5, http://links.lww.com/MR/A330), supporting an association between cancer burden and ctDNA levels; however, we did not observe a significant difference in the percentage of patients showing ctDNA detection at baseline between high and low metastatic load (*P* = 0.4, Supplementary Figure 4B, Supplemental digital content 5, http://links.lww.com/MR/A330).

### Patients resistant to treatment harbor *TERT* alterations in circulating tumor DNA

Next, we determined the percentage of patients with detectable mutations annotated as drivers in ctDNA in any of the 40 panel genes (methods). Of the 40 genes investigated, we found at least one driver mutation in 20 genes in at least one patient. We observed that the telomerase gene *TERT* was the most commonly altered gene found mutated in ctDNA in patients that developed therapy resistance (affecting 5/13 resistant and acquired resistant patients). In comparison, only 1/11 patients with response to therapy harbored a mutation in *TERT* (Fig. 2c and Supplementary Figure 4C, Supplemental digital content 5, http://links.lww.com/MR/A330). This is consistent with findings from previous studies associating *TERT* with poor outcomes [33] and implicates *TERT* with immunotherapy resistance. This gene was found as most commonly altered in the high metastatic load subgroup as well, compared to their low metastatic load counterparts (Fig. 2d and Supplementary Figure 4D, Supplemental digital content 5, http://links.lww.com/MR/A330). We identified a similar trend when comparing deceased and alive patients, with *TERT* being the most frequently mutated gene in the deceased subgroup (affecting 4/14 deceased and 2/10 alive patients, Supplementary Figure 3C, Supplemental digital content 4, http://links.lww.com/MR/A329). *BRAF* was the second most mutated gene overall, affecting 3/9 resistant patients and 2/11 responders. No significant difference in the frequency of *BRAF* mutations was observed between responders and resistant patients. When we compared patients with resistance to patients with acquired resistance, we observed no pattern in the overall mutations found in ctDNA arising after initiation of immunotherapy (Fig. 2e, red squares indicate mutations only found in later liquid biopsies). Thus in this limited cohort, no mutation to specific genes could be associated with acquired resistance to immunotherapy.

### Patients with response to therapy tend to harbor fewer detectable driver mutations in circulating tumor DNA

**Fig. 3:**
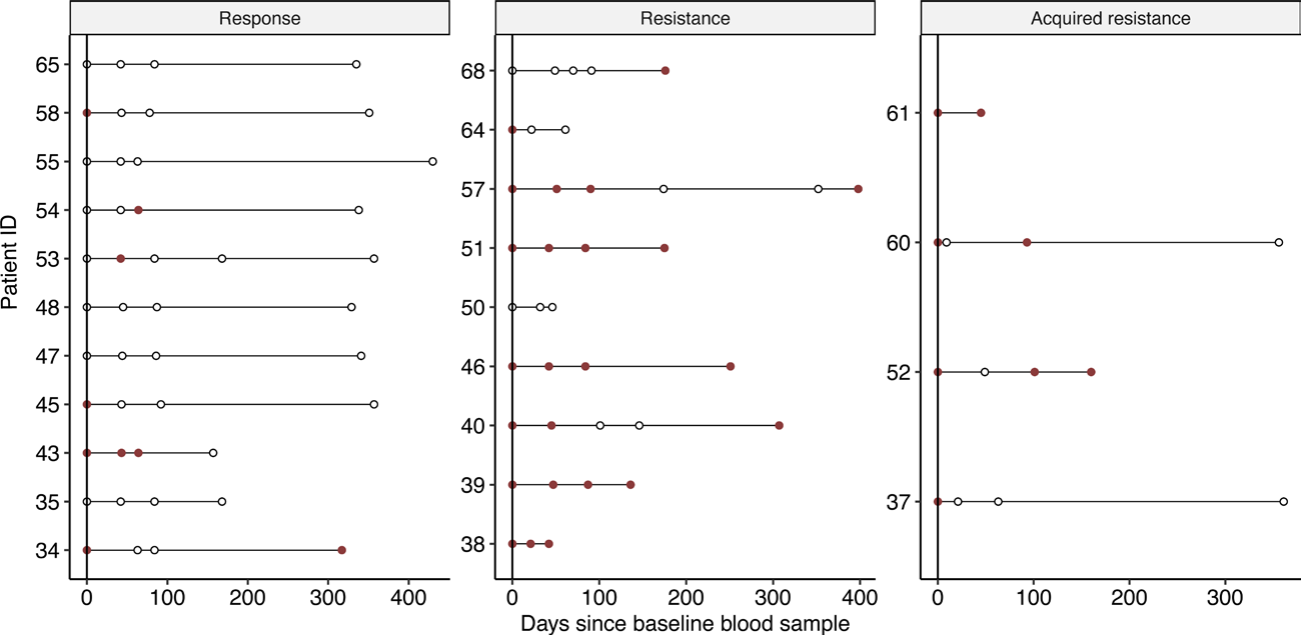
Driver mutation detection per response category. Dotplots showing driver mutation detection status over the course of the study. Y-axis shows patient ID, X-axis shows the days passed since the baseline blood sample was extracted. Dots correspond to blood tests taken, empty dots indicate no driver mutation detected, red dots indicate that driver mutations were detected in the sample.

When we compared ctDNA between the patient response groups, ctDNA was detected at baseline in 4/11 (36%) of the responder patients. This compares to 7/9 (78%) and 4/4 (100%) of the resistant patients and patients showing acquired resistance, potentially reflecting a lower initial disease burden among patients responding well to therapy (Fig. 3). As expected, all responding patients showed a decline in ctDNA during their treatment, with no patients having detectable ctDNA in their last blood sample. In comparison, ctDNA was found in the last blood sample of 7/9 resistant patients. Patients 50 and 64 were both resistant to therapy but showed no ctDNA in their last blood sample. No ctDNA was found at any time point for patient 50, which may indicate that the cancer harbored no driver mutations within the panel, making it essentially undetectable by our tumor-agnostic panel. Conversely, a single-driver mutation, the chr12 : 45852805:T alteration affecting *ARID2*, was found in patient 64 at baseline. This mutation was not found during follow-up and may represent a minor subclone eliminated by the treatment. Among the patients with acquired resistance, 4/4 showed at least one ctDNA-positive blood sample during follow-up. Patient 37 showed detectable ctDNA only at baseline, despite recurrence detected by CT scan prior to the last blood sample being obtained. This may reflect an overall low disease burden or subclonal selection resulting in outgrowth of a subclone harboring no driver mutations in the ctDNA gene panel. Considering resistant and acquired resistance patients together, we observe that overall 12/13 patients had at least one ctDNA positive sample, indicating that the current tumor-agnostic gene panel, selected to enrich in known cancer genes commonly mutated in melanoma, can detect cancer in 92.3% of patients. In this analysis, patients showing a response to therapy are not included, as we here cannot discern between cancers that are negative due to no driver mutations found within the ctDNA gene panel and cancers that are negative due to therapeutic response to therapy.

It has previously been suggested that ctDNA detection likely mostly depends on the total number of cancer cells [34], which is consistent with the finding that patients with a higher metastatic load showed a higher MAF on average (Supplementary Figure 4A, Supplemental digital content 5, http://links.lww.com/MR/A330); however, in this cohort of limited size, metastatic load showed no association with outcome (Supplementary Figure 5, Supplemental digital content 6, http://links.lww.com/MR/A331).

### Evaluating circulating tumor DNA dynamics during therapy

**Fig. 4:**
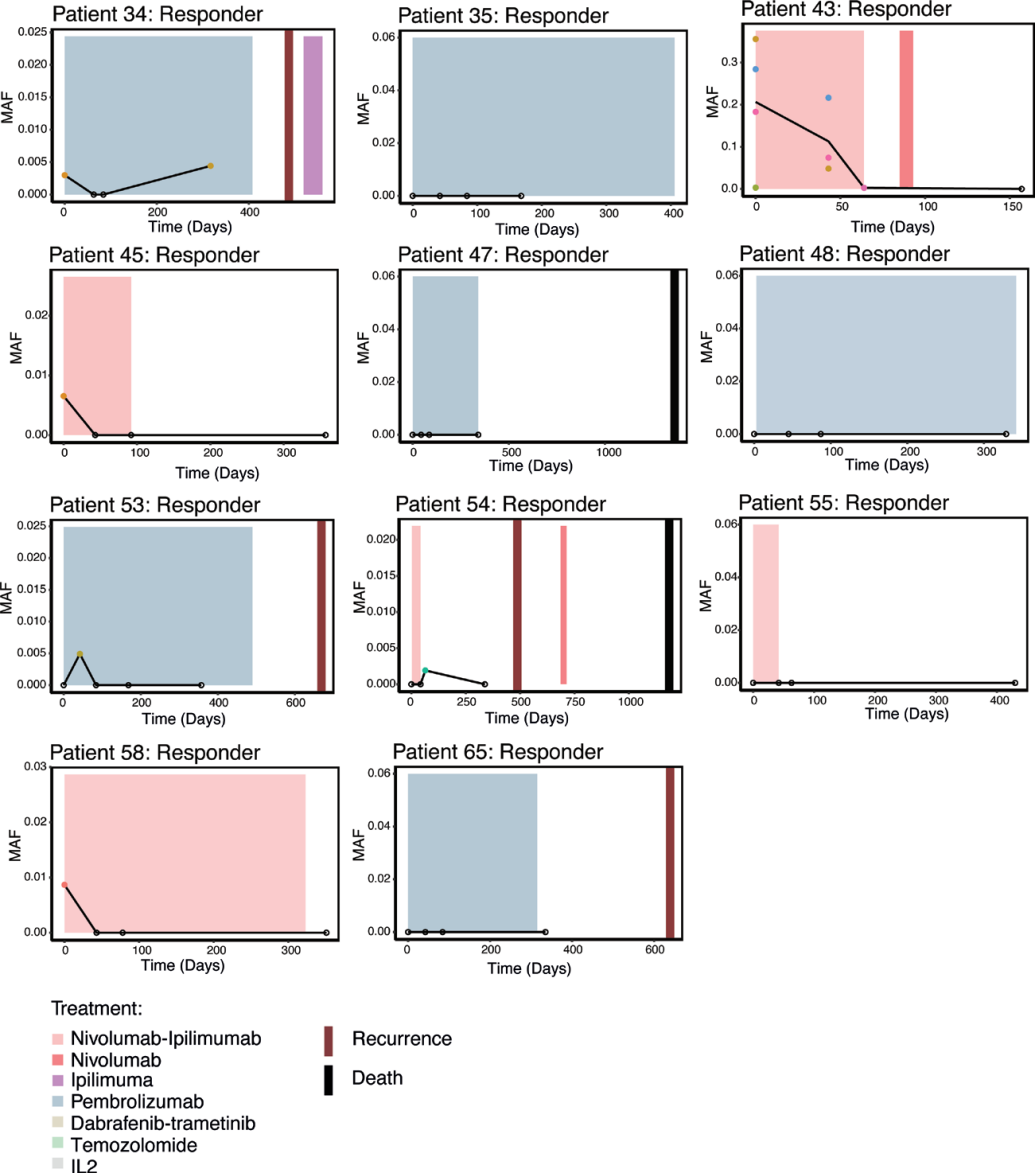
Longitudinal analysis of ctDNA in patients with response. Per-patient plots showing the driver mutations detected over the course of treatment and clinical history. Y-axis shows the mutant allele frequency, X-axis shows the days since the baseline blood sample. Colored dots indicate detected mutations, empty dots signify that a blood sample was taken at the time point but no mutation was detected. Black line connecting the time points corresponds to the mean MAF over time. Colored boxes show the type and time frame of treatment. Red vertical lines show the date of clinical progression, black vertical lines show the date of death. Dashed vertical lines show the date of PET/CT or CT scans. CT, computed tomography; ctDNA, circulating tumor DNA; MAF, mutant allele frequency.

**Fig. 5:**
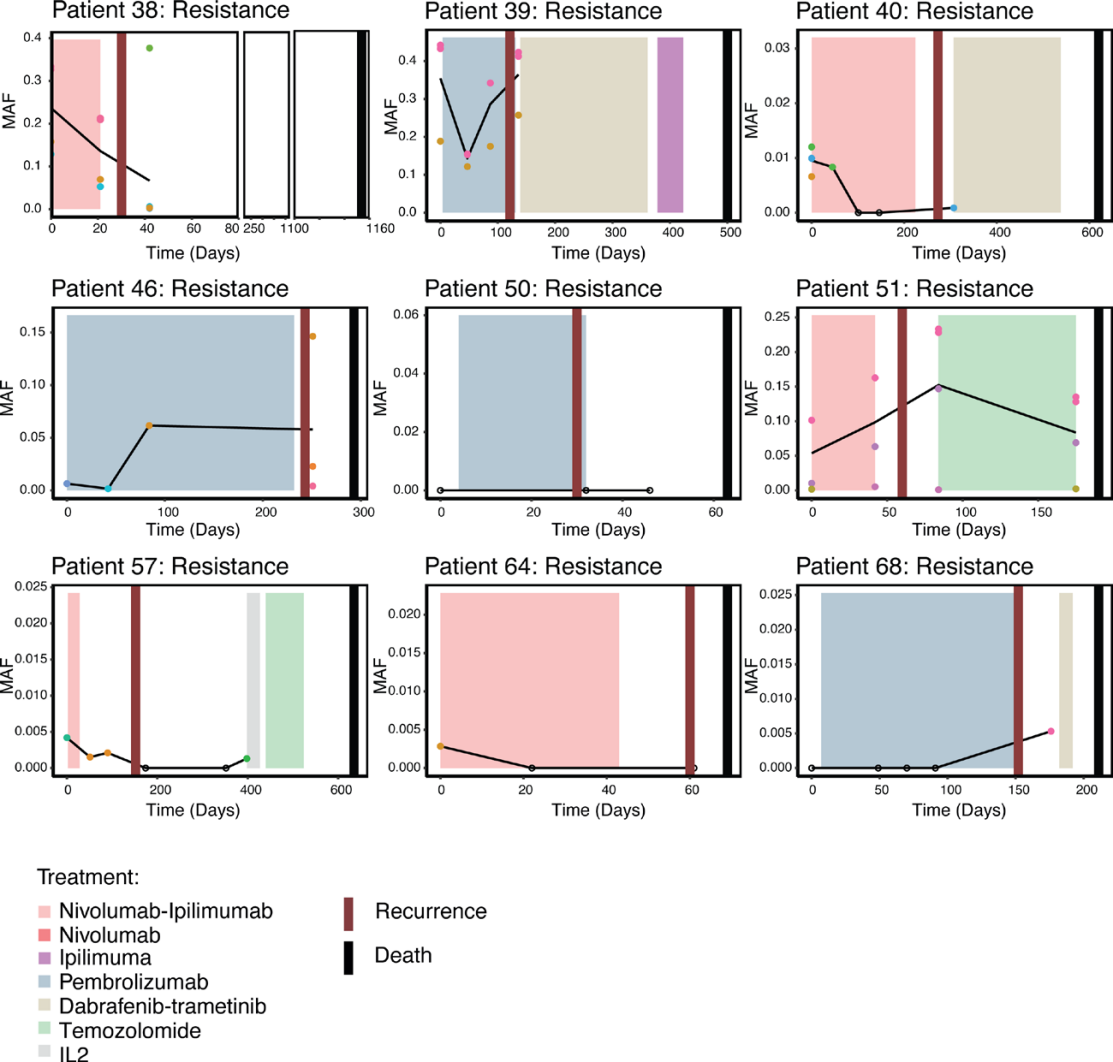
Longitudinal analysis of ctDNA in patients with resistance. Per-patient plots showing the driver mutations detected over the course of treatment and clinical history in patients resistant to treatment. Annotation follows Fig. 4. ctDNA, circulating tumor DNA.

**Fig. 6:**
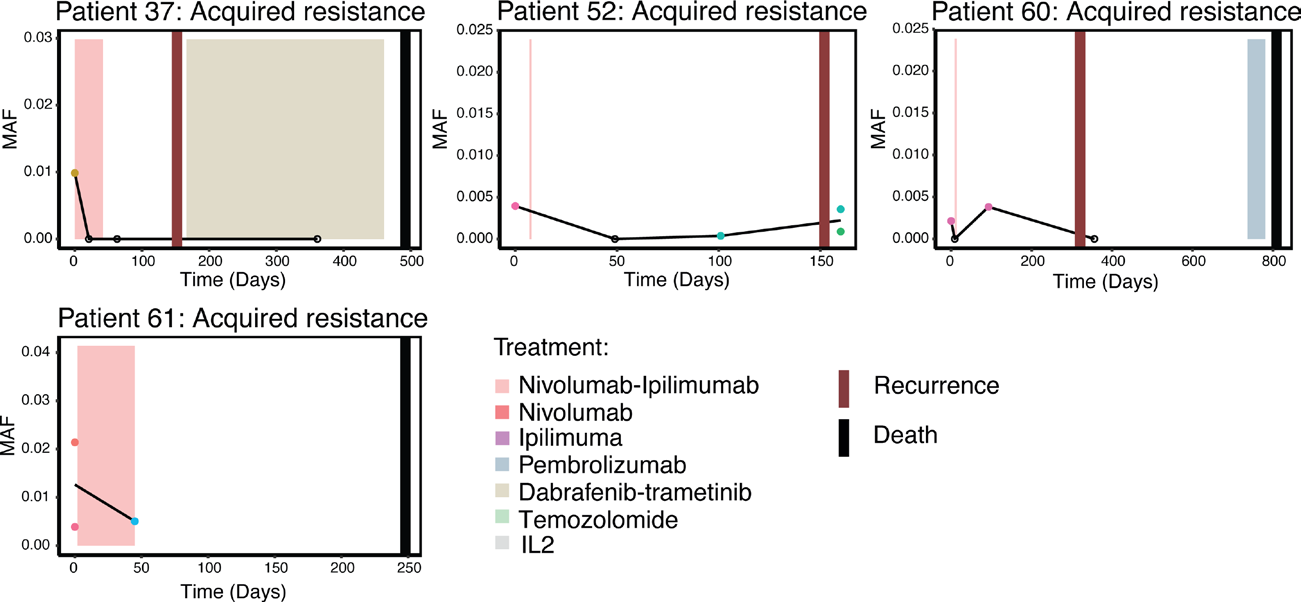
Longitudinal analysis of ctDNA in patients who acquired resistance. Per-patient plots showing the driver mutations detected over the course of treatment and clinical history in patients developing acquired resistance to treatment. Annotation follows Fig. 4. ctDNA, circulating tumor DNA.

Lastly, we investigated the utility of the ctDNA panel as a biomarker for treatment response in a longitudinal setting. For this purpose, we analyzed the ctDNA MAF in serial samples in all patients and compared it to treatment response (Figs. 4–6). In patients with response to treatment, ctDNA was not detected at any time point in 5/11 patients. In two patients, we detected ctDNA at baseline only. In one patient, patient 43, ctDNA was detected at baseline and decreased in MAF while on treatment, falling below the detection limit as treatment was discontinued (Fig. 4).

In patients showing treatment resistance, ctDNA was detected in 7/9 patients at baseline. One additional patient became ctDNA positive as their disease progressed. We observed an increase in ctDNA MAF between baseline and clinical relapse for 4/9 patients (Fig. 5).

For patients who acquired resistance to treatment, ctDNA levels were low, but were detected at baseline for all four patients (Fig. 6). Overall, these observations indicate that in this cohort, ctDNA dynamics alone cannot be used as a reliable biomarker of therapeutic response.

## Discussion

In this study, we report differences in genomic alterations between patients that have a complete response to immunotherapy compared to patients that have either no response or have developed acquired resistance. By using a unique panel of well established genes known to be involved with the development of melanoma and CPI response, we have demonstrated how genomic data can be used to analyze and identify certain differences in responses to immunotherapy in patients with metastatic melanoma. Consistent with current literature, we have found that a higher percentage of resistant and acquired resistance patients harbor a mutation in *TERT* compared to their responder counterparts. While we cannot exclude that *TERT* mutations may also be found in subclones in tumors not shedding ctDNA, our data indicate that genomic alterations in the *TERT* gene found in ctDNA can be used as a predictor for poor prognosis and poor response to immunotherapy. Currently, there is a strong focus on investigating ctDNA and exploring and validating the use of ctDNA in clinical practice. One of the strengths of our study is the continuous blood samples obtained during treatment, which has enabled analysis of the dynamics of ctDNA over time. Potentially, continuous blood samples can be used to detect recurrence even before the cancer is detectable on follow-up scans. We observed no differences in overall ctDNA levels between responders, resistant, and acquired resistant patients in this cohort, either at baseline or at any time point during treatment and follow-up. This indicates that ctDNA levels alone may not be sufficient to identify metastatic melanoma patients likely to respond to immunotherapy; however, other studies have demonstrated a correlation between low levels of ctDNA and disease burden, also in metastatic melanoma patients [35]. Thus, our results may indicate a sensitivity issue with tumor-agnostic approaches such as the one applied here. Particularly, the panel gene set was limited to 40 genes, representing a relatively small panel size, which limited sensitivity. Since our study commenced, further experience and technical improvements in ctDNA purification methods have demonstrated improved yields. Particularly, it is today standard to double-spin samples prior to plasma collection as this reduces contaminating nuclear DNA [36]; however, our study was initiated before this was established as a superior methodology, and to ensure uniformity in sample collection, all samples were only subjected to a single round of centrifugation. This may have reduced the total ctDNA yield per sample, and thus negatively affected our ability to detect somatic mutations, particularly in samples with low ctDNA burden. Despite these limitations, we did observe using our tumor-agnostic panel that resistant and acquired resistant patients tended to be ctDNA positives at baseline more often than responders.

In our work, we found significant differences in ctDNA MAF when we compared patients with high and low metastatic loads. This indicates that, in line with current literature, patients harboring higher metastatic load will shed more ctDNA into circulation due to a higher cancer cell burden [34]. Additionally, we observed an enhanced signal of *TERT* mutations in the high-load group, which is consistent with already published work [33] associating *TERT* with poor prognosis. Potentially, *TERT* can act as a biomarker for identifying patients likely resistant to immunotherapy, however, this needs to be further validated in a larger cohort.

A major limitation to our study is the small cohort size as well as a lack of tumor biopsies or germline control samples which is a challenge for ctDNA mutation calling and makes it difficult to evaluate the performance of our variant filtering and noise reduction. While we use independent samples as background for variant calling and known SNP databases to filter out normal alterations, we expect that some melanoma-specific variants remain undetected or are excluded during filtering. Nevertheless, after meticulous analysis of the data, we here show how a tumor-agnostic panel ctDNA can be used to inform about tumor biology and cancer progression, and we believe our study may serve as a starting point for deeper investigations into the utility of ctDNA in metastatic melanoma and the biology of treatment response.

## Acknowledgements

The study (#1-10-72-230-19) received ethical approval on 21 January 2021. The committees on Biomedical Research Ethics in the Central Region of Denmark approved the study (#1-10-72-230-19). The study was performed in accordance with the Declaration of Helsinki and all patients provided written informed consent. The data generated in this study are available within the supplementary data files. Due to privacy laws, access to raw sequencing data is restricted. Raw data can therefore only be made available following approval from the Danish National Committee on Health Research Ethics and the Danish Data Protection Agency. Access requests should be directed to the corresponding author.

### Conflicts of interest

There are no conflicts of interest.

## Supplementary Material












