# Supplementary figures and images for: Analysis of circulating tumor DNA during checkpoint inhibition in metastatic melanoma using a tumor-agnostic panel
Source: Melanoma Res. 2023 Jun 12;33(5):364–74. doi: 10.1097/CMR.0000000000000903 (PMC10470440; doi:10.1097/CMR.0000000000000903)

# Supplementary Figure 1

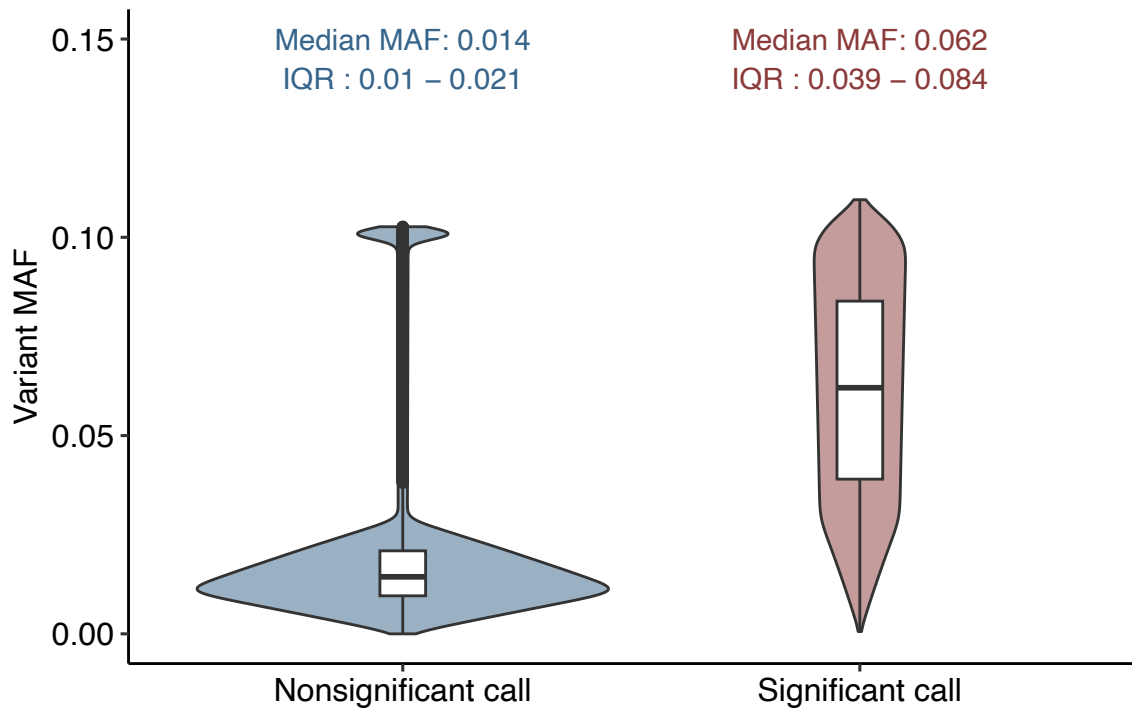

Supplement: Supplementary file 2 [file mr-33-364-s002.pdf]

Supplementary Figure 2

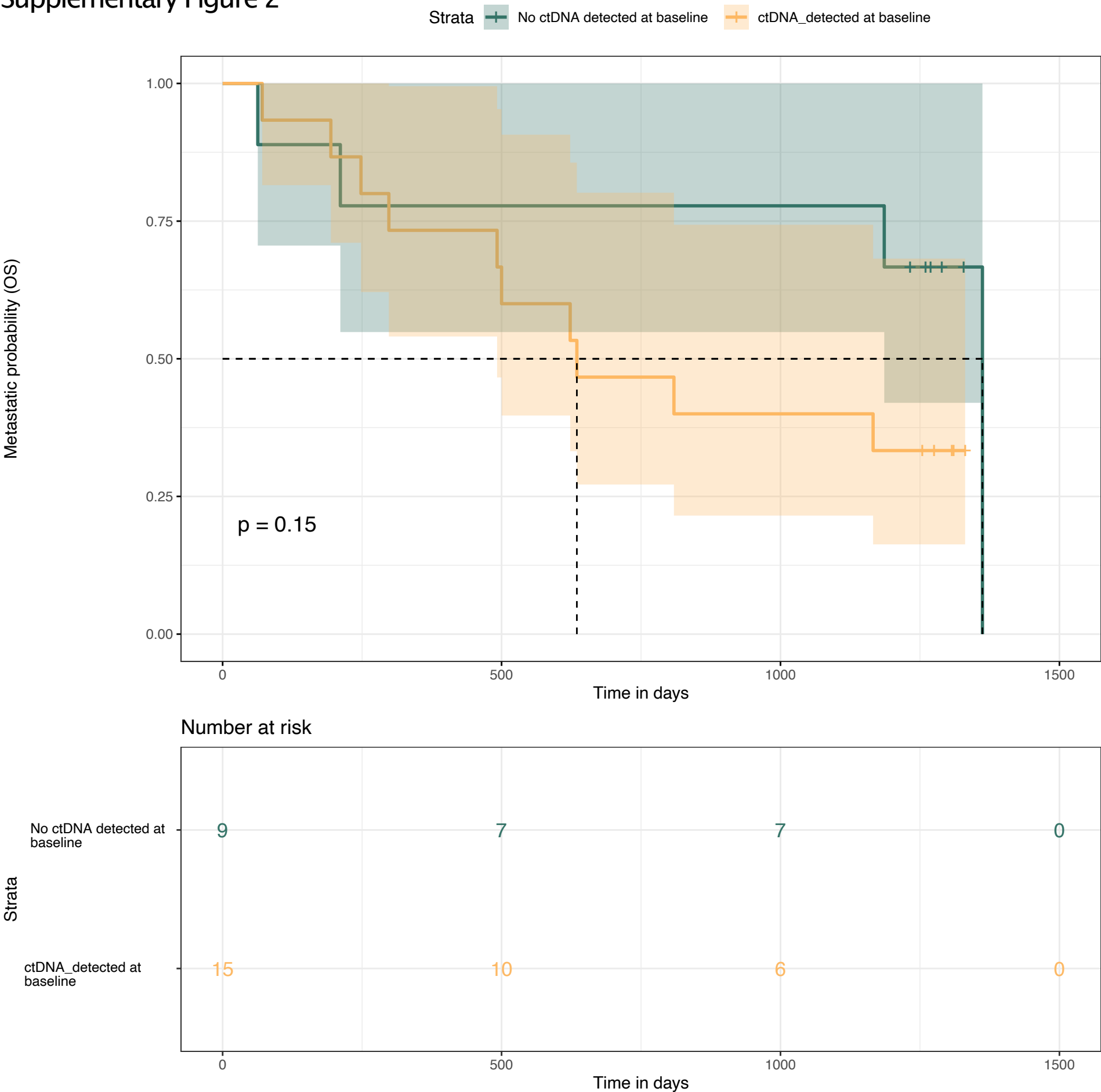

Supplement: Supplementary file 3 [file mr-33-364-s003.pdf]

Supplementary Figure 3

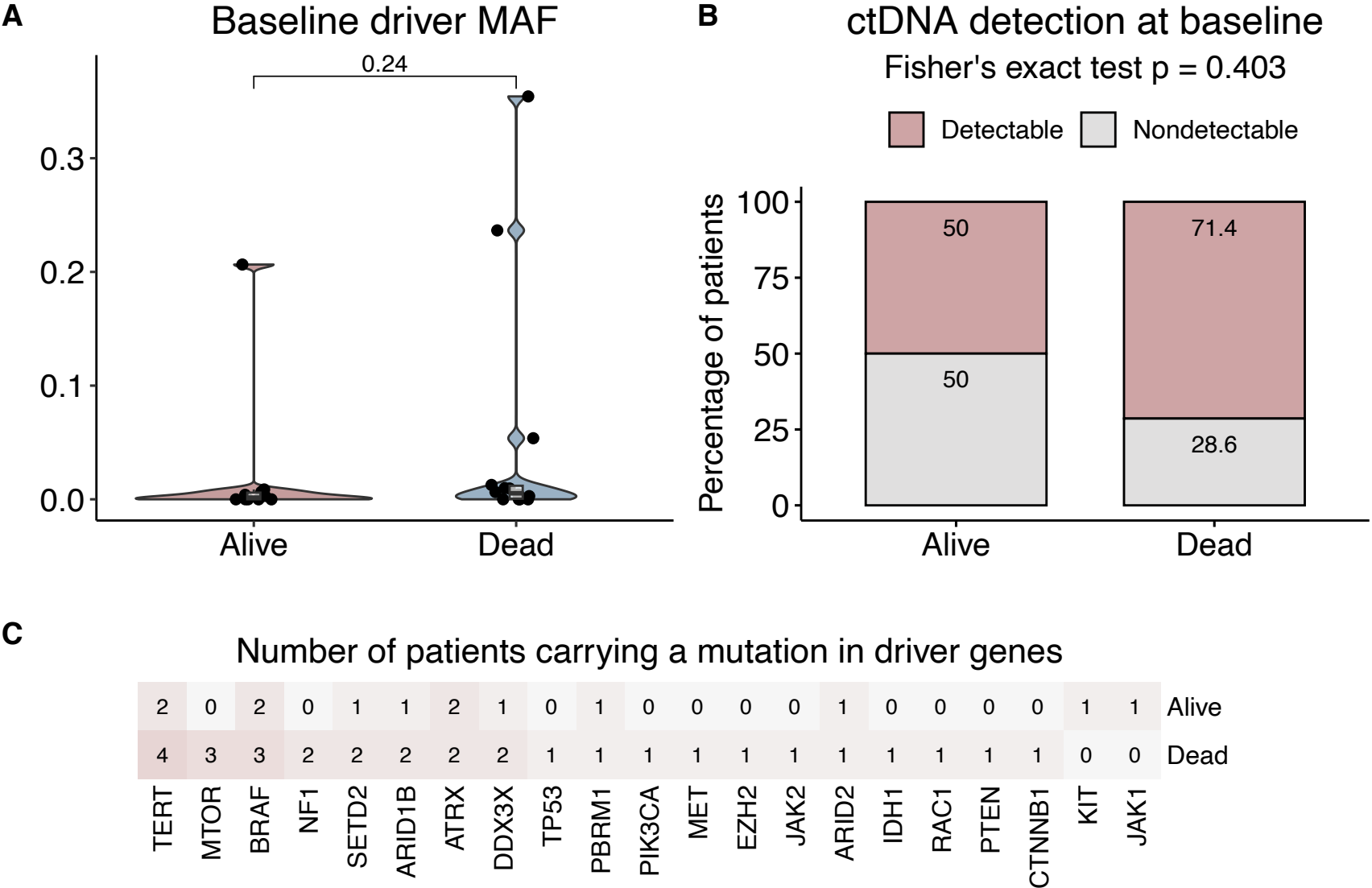

Supplement: Supplementary file 4 [file mr-33-364-s004.pdf]

## Supplementary Figure 4

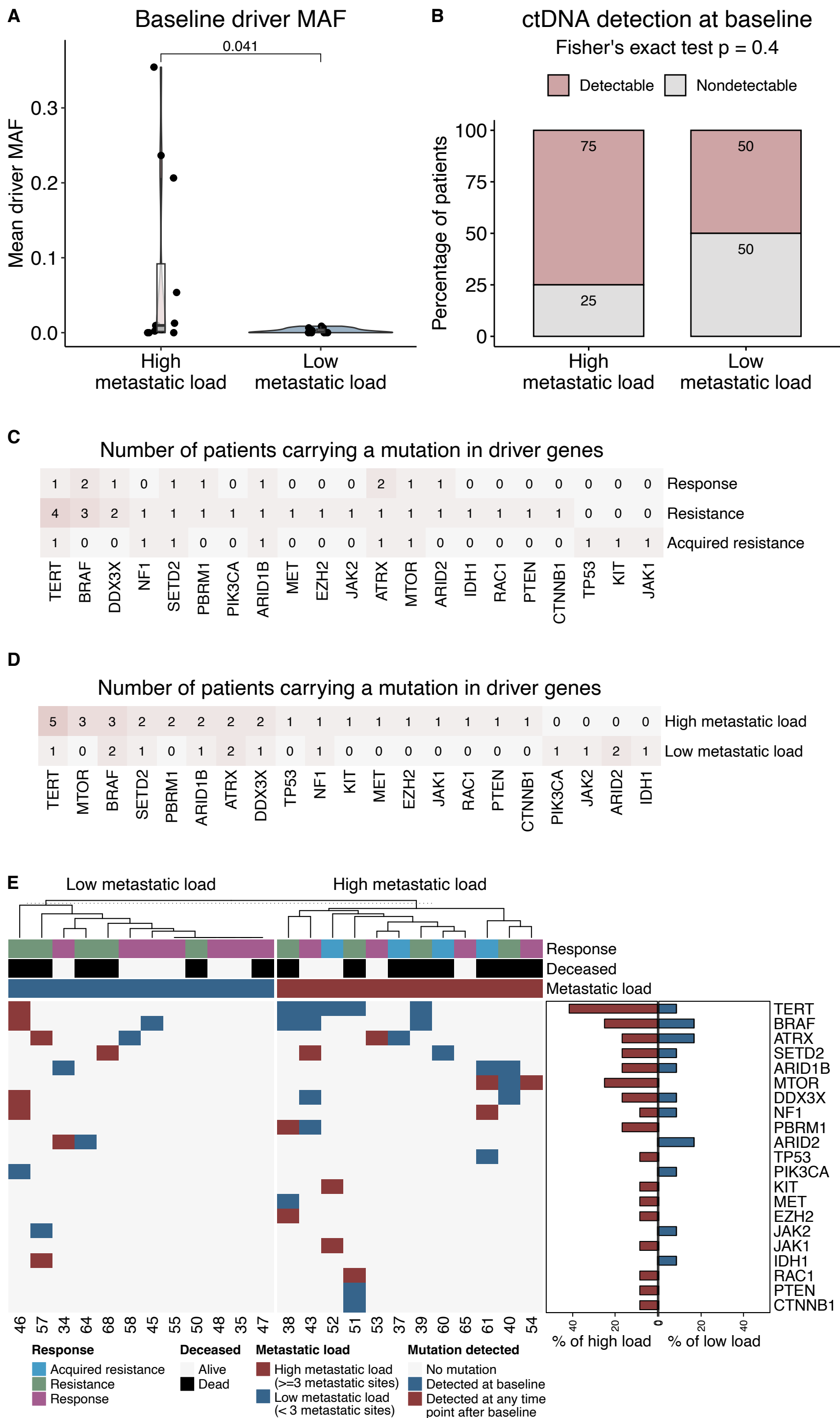

Supplement: Supplementary file 5 [file mr-33-364-s005.pdf]

Supplementary Figure 5

Strata 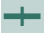 Low metastatic load 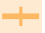 High Metastatic load

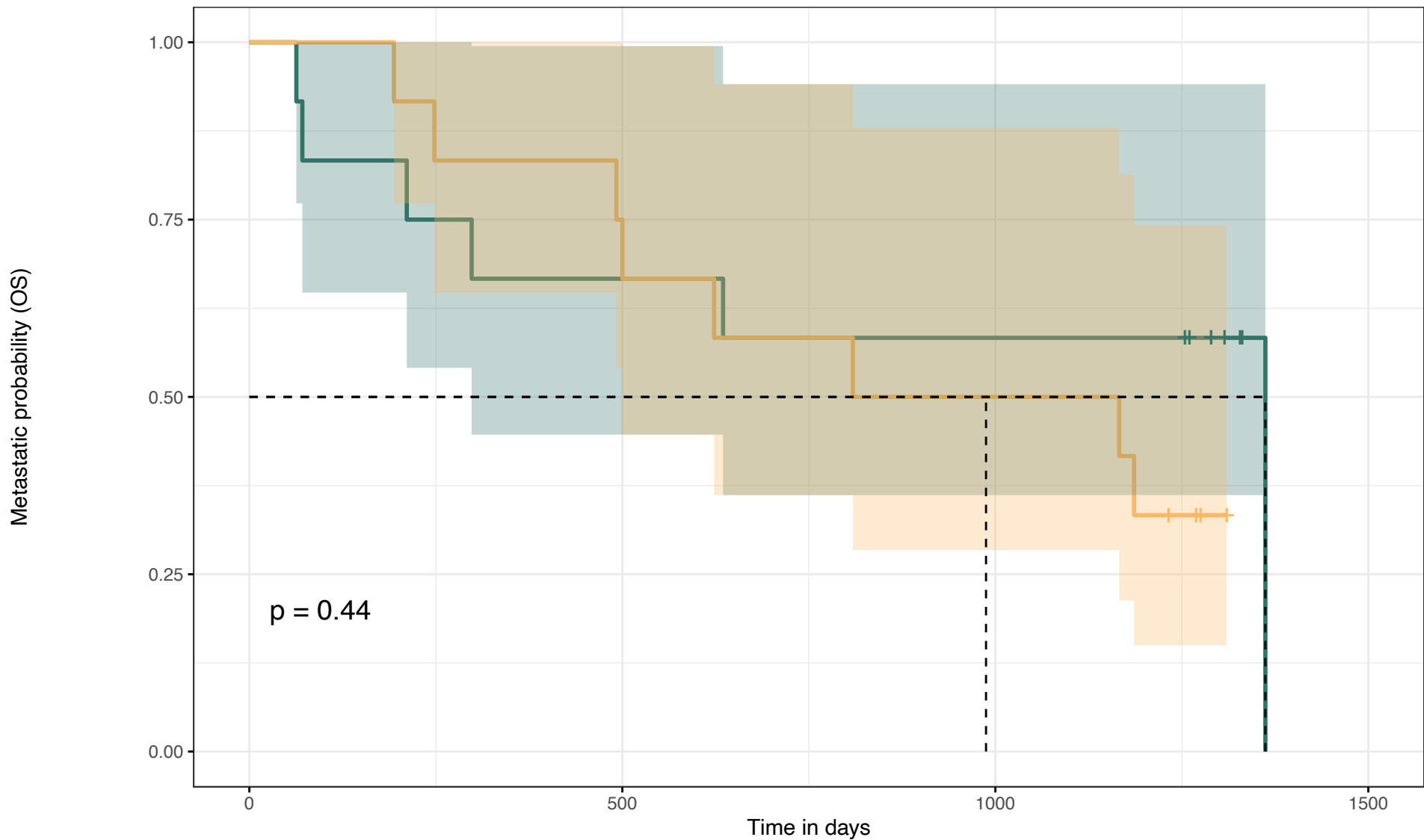

Number at risk

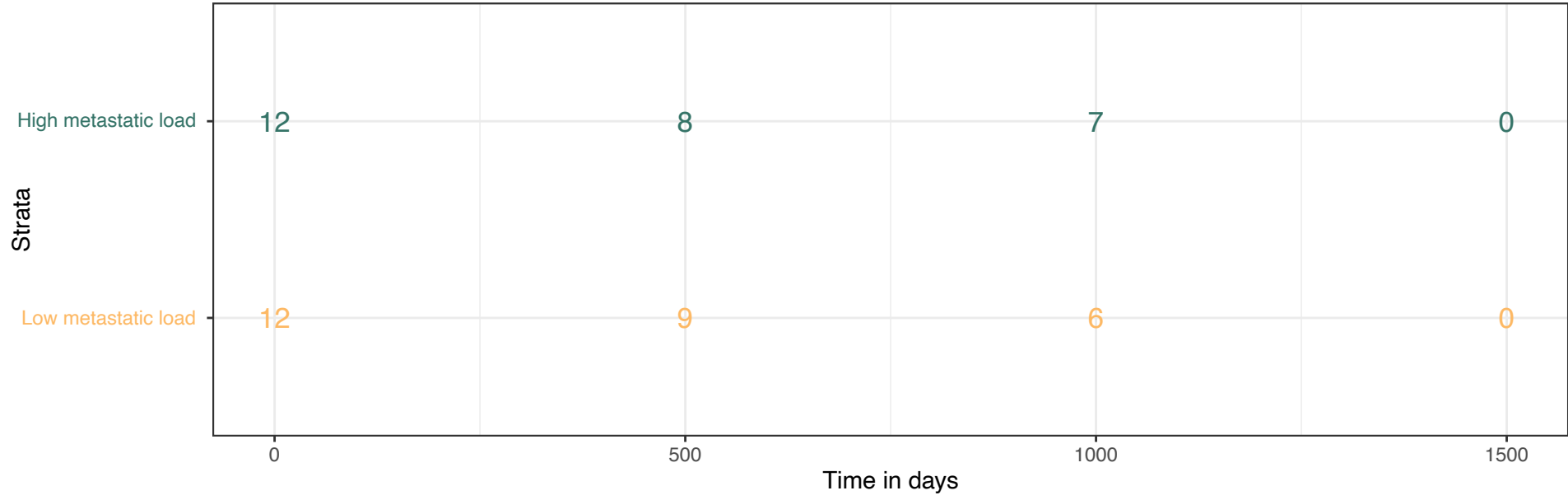

Supplement: Supplementary file 6 [file mr-33-364-s006.pdf]
